# Effect of Light Curing Distance on Microhardness Profiles of Bulk-Fill Resin Composites

**DOI:** 10.3390/polym14030528

**Published:** 2022-01-28

**Authors:** Fatin A. Hasanain, Hani M. Nassar, Reem A. Ajaj

**Affiliations:** Department of Restorative Dentistry, Faculty of Dentistry, King Abdulaziz University, Jeddah 21589, Saudi Arabia; hnassar@kau.edu.sa (H.M.N.); raajaj@kau.edu.sa (R.A.A.)

**Keywords:** microhardness, bulk-fill composites, microhardness ratio

## Abstract

Bulk-fill (BF) dental resin composites are made to be polymerized in increments of up to 5 mm rather than the 2 mm increment recommended for conventional composites. This project aimed to determine microhardness (MH) profiles of BF resin composites at different depths and varying light cure (LC) distances from the light source in an attempt to mimic varying clinical situations. Forty-eight cylindrical specimens (4 mm diameter and 6 mm height) were prepared from 3 BF composites: Tetric N-Ceram Bulk-Fill (TBF), Filtek One Bulk-Fill (FBF), and Sonic-Fill 2 (SF2). Four different distances (0, 2, 4, and 6 mm) from the LC unit were investigated. Vickers MH was measured at the top and bottom of the samples and at every 1 mm, by creating 3 indentations at each depth. The bottom-top microhardness ratio (MHR) and percentage reduction in MHR were also measured. Data was analyzed using mixed-model repeated-measure ANOVA at 0.05 significance level. The main variables effects “material, LC distance, and depth” were significant (*p* < 0.001). Increasing LC distance and the depth of the tested BF significantly affected Vickers MH and MHR. None of the tested BF materials had sufficient MHR at the depths of 4–6 mm. SF2 showed the least MHR reduction.

## 1. Introduction

Dental resin composites are the direct filling material of choice for many clinicians due to their pleasing esthetics and good mechanical properties [1,2,3]. These materials are essentially filled resins that polymerize to form hard filling materials in or on the tooth structure [4]. The polymerization process is initiated by visible light and requires deep light penetration to allow for optimum polymerization and mechanical properties [5]. One of the most common photoinitiators, camphorquinone, has maximum absorption at a wavelength of 468 nm [5,6]. However, filler particles scatter the light, which affects the depth of light penetration. To ensure sufficient light penetration and curing despite the previously mentioned limitations, most conventional dental resin composite materials must be placed in increments of 2 mm or less, which increases the time required for placement as well as the sensitivity of the restorative technique [1]. In an effort to improve upon the existing materials, several bulk-fill dental resin composites were introduced by manufacturers. These materials claim to be curable in 4- to 5-mm increments while adhering to the same exposure time recommended for conventional composites [7,8]. In theory, this is made possible by one of the following methods; reducing the filler content to create a flowable composite, by changing the photoinitiator, or by increasing the translucency of the material [1]. These changes allow the light to penetrate deeper into the restoration.

Adequate polymerization, initiated by light curing, is vital for dental resin composite restorations. The clinical performance of the final restoration is affected by the efficiency and light quality of the light cure device used by the clinician [9]. Incomplete polymerization can lead to a variety of negative sequelae, which include, but are not limited to, discoloration of the restoration, post-operative sensitivity, and pulpal irritation, as well as cytotoxicity [10,11]. Moreover, deterioration at the margins along with a reduction in hardness and bond strength could take place [10]. This will eventually lead to restoration failures.

It is advised that clinicians place their light cures as close to the restoration surface as possible to maximize polymerization and obtain the optimum properties [12,13,14]. However, that is not always possible in clinical practice. The floor of the cavity is further away from the light cure tip than the occlusal surface, which will result in a further curing distance from the composite surface [15]. This distance increases even more in deep proximal cavities. Some dentists also inadvertently hold the light cure away from the restored surface, resulting in a 2–3 mm gap between the light cure tip and the restored surface. All these factors adversely affect the final properties of a restoration, which has a recommended curing depth of 2 mm. Given all these considerations, the introduction of bulk-fill dental resin composite was welcomed by most practitioners [16].

It has been estimated that approximately 5000 composite restorations are placed annually at a single teaching dental institution by undergraduate students alone [17]. Using bulk-fill materials will save time and effort and enable the staff and students to provide a high level service to an even larger number of the population. This will follow into dental practices as well. However, the question remains of whether or not the bulk-fill restorative materials are adequately cured when placed in single increments of 4–5 mm rather than the much smaller 2 mm increments used with conventional composite restorations.

Other projects have looked at the microhardness of bulk-fill dental composites with varying results [18,19,20]. Some of them have focused on the microhardness from a single distance from the light source, while others have examined it with different distances from the light source. In a similar vein, this project will focus on determining the microhardness profiles of bulk-fill dental resin composites at different depths and with varying distances from the light source. It will, however, test the materials beyond the manufacturers’ recommended depths, as that is a likely clinical scenario. It will also measure the spectral irradiance from the light curing unit to determine the actual irradiance value at each of the tested distances. In light of these findings, recommendations can then be made for the most appropriate use of bulk-fill restoratives. The null hypothesis for this project was that there is no significant difference between the materials tested, regardless of their depth or distance from the light cure.

## 2. Materials and Methods

A total of 48 specimens were prepared using 3 different bulk-fill composites at 4 different distances from the light curing unit (LC). Each material had 4 different groups made; at 0, 2, 4 and 6 mm away from the LC. The materials used are Filtek One Bulk-Fill (FBF), Tetric N-Ceram Bulk-fill (TNB), Sonic Fil 2 (SF2), and shown in Table 1.

**Figure 1:**
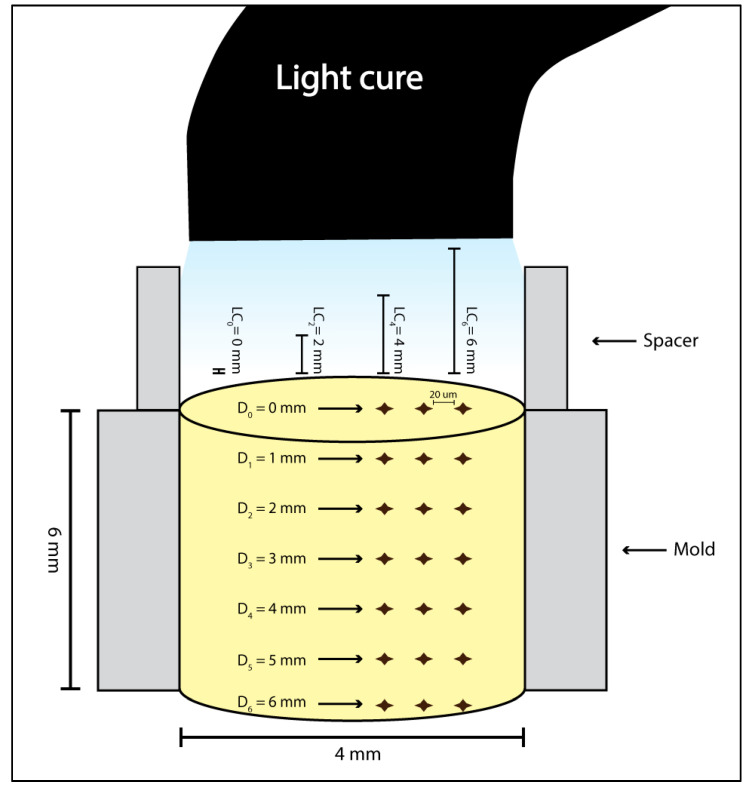
A Diagrammatic illustration of the specimen setup showing the dimensions of the specimen, light cure distances, and indentation arrangement according to depths.

A cylindrical stainless-steel split mold with the internal dimensions of 4 mm in width and 6 mm in height (New Age Research, USA) was used to prepare the samples. The mold was placed on top of a Mylar strip and glass slide. The test material was then placed into the mold as a single increment to mimic the clinical scenario. Another Mylar strip and glass slide were placed on top of the filled mold. The glass slide was then removed, and the material light cured for 20 s by a LED LCU (3M ESPE Elipar, St Paul, MN, USA). The power density was measured with a handheld radiometer (Bluephase Meter II, Ivoclar, Amherst, NY, USA) at 1200 mW/cm^2^ immediately before specimen preparation. This was the setup at 0 mm away from the LC. For each of the remaining distances, the same initial setup was used but the glass slide was replaced with a stainless steel split mold with an internal diameter of 2, 4, or 6 mm in height and 4 mm in width. This would allow the LC to be held at a set distance away from the sample. The resultant sample groups were demarcated as LC_0_, LC_2_, LC_4_, and LC_6_. Figure 1 illustrates the sample preparation setup, complete with spacers when used.

Upon completion of the sample preparation, the samples were stored in the dark for 1 week to allow full polymerization. Microhardness (MH) was then measured at the top (D_0_) and bottom (D_6_) surfaces of the samples and at every 1 mm in between at the side of the stabilized sample using a Vickers hardness tester (ZHV30, Zwick/Roell, Ulm, Germany) with 100 g load for 10 s.

To determine the reduction of MH due to the increase in LC distance, absolute and relative reductions at each depth were calculated in relation to LC_0_. The absolute reduction in MH was calculated by subtracting the value at LC_2_, LC_4_, and LC_6_ from values at LC_0_. The relative reduction in microhardness ratio (MHR) was determined by calculating the percentage decrease in DC at each LC compared to LC_0_.

All the data was collected, tabulated, and subjected to statistical analysis. Statistical analysis was performed by SPSS (version 20). Microsoft Office Excel 2010 was used for data handling and graphical presentation.

For MH as an independent variable, univariate analysis general linear model (GLM) procedure with three fixed factors: material, depth, and LC distance is applied. A Post hoc test for multiple comparisons by Bonferroni method was applied. A similar analysis was used for MHR as an independent variable.

One way analysis of variance (ANOVA) was applied for comparing microhardness at the same depth and material but with different LC distances. A Post hoc test for multiple comparisons using Bonferroni method was applied. Significance level was set at *p* < 0.05.

Spectral irradiance was measured by creating 12 custom rectangular glass spacers with the following dimensions: 10 mm width, 1 mm depth, and 20 mm length. The glass spacer was placed on the radiometer and the irradiance measured 3 times. The spacers were stacked to simulate the distance between the LC unit and restorative surface in single mm increments.

## 3. Results

### 3.1. Vickers Microhardness

Means and standard deviations of tested bulk-fill materials at different depths and LC distances are shown in Table 2. The main effects of all included variables (material, LC distance, and depth) are significantly different (*p* < 0.001). The interaction effects “material” × “depth” and “LC distance” × “depth” were also significantly different (*p* < 0.001). TBF was significantly different from FBF and SF2 (*p* < 0.001). FBF and SF2 were not significantly different from each other (*p* = 0.758). Microhardness values were significantly different (*p* < 0.001) at all light cure distances except between 2 and 4 mm (*p* = 1.00). Microhardness values were significantly different (*p* < 0.001) at all depths except between 0 and 1 mm (*p* = 1.00).

### 3.2. Microhardness Ratios

**Figure 2:**
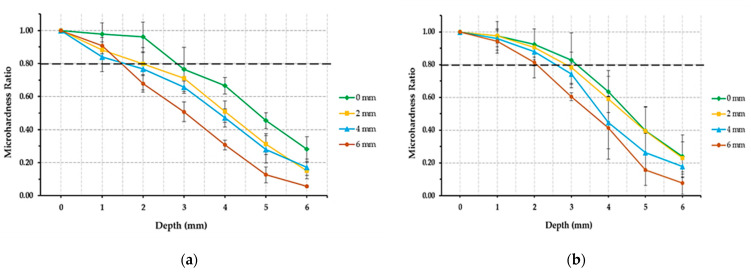

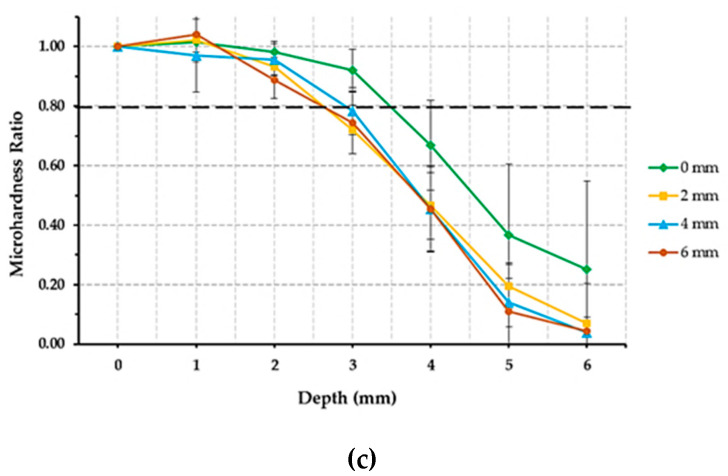
Line graphs showing microhardness ratio values of the different bulk-fill materials at different depths and light cure distances. (**a**) Tetric N-Ceram BF, (**b**) Filtek One BF, and (**c**) Sonic Fill 2. Error bars represent standard deviations. All values were significantly different (*p* ≤ 0.05) from each other expect 0 compared to 1 mm depth. All values were significantly different (*p* ≤ 0.05) from each other expect 2 compared to 4 mm light cure distance. Dashed lines represent acceptable microhardness ratio (≥0.8) according to Poggio et al. [21].

**Figure 3:**
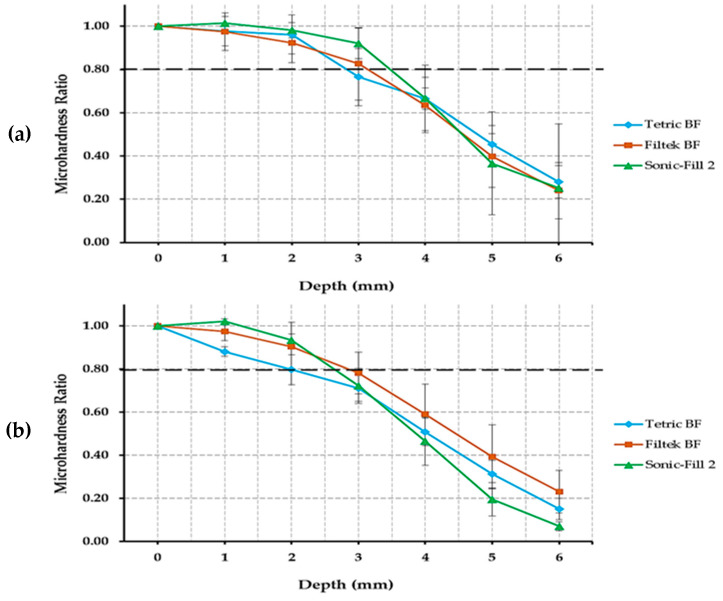

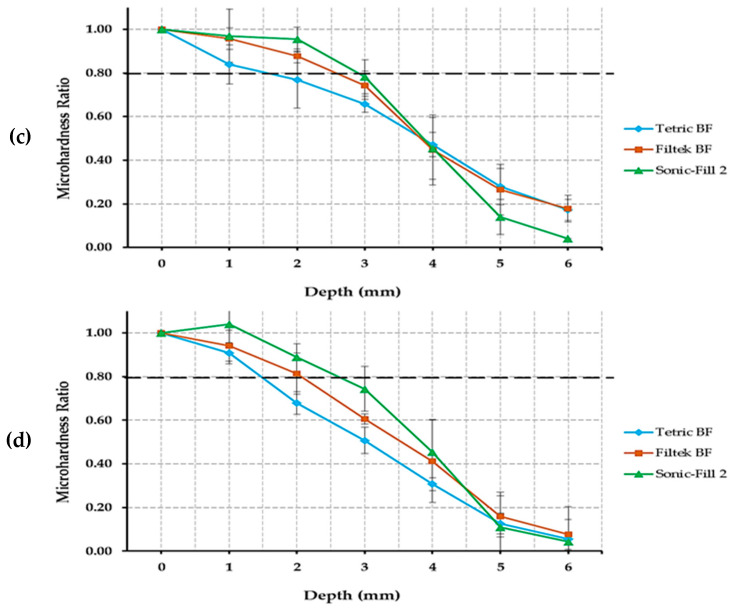
Line graphs showing microhardness ratio values of the different bulk-fill materials at different light cure distances: (**a**) 0 mm, (**b**) 2 mm, (**c**) 4 mm, and (**d**) 6 mm. Error bars represent standard deviations. Tetric N-Ceram BF was significantly different from Filtek One BF and Sonic-Fill 2 (*p* ≤ 0.05). Filtek One BF was non-significantly different from Sonic-Fill 2 (*p* > 0.05). Dashed lines represent acceptable microhardness ratio (≥0.8) according to Poggio et al. [21].

The means of MHRs are shown in Figure 2 and Figure 3. The main effects of all included variables (material, light cure distance, and depth) are significantly different (material, *p* = 0.001; light cure distance and depth, *p* < 0.001). All interaction effects were also significantly different “material” × “LC distance” *(p* = 0.004), “material” × “depth” and “LC distance” × “depth” *(p* < 0.001). TBF was significantly different from FBF (*p* = 0.003) and SF2 (*p* = 0.01). FBF and SF2 were not significantly different from each other in regard to MHR (*p* = 1.00). MHR values were significantly different (*p* < 0.001) at all light cure distances except between 2 and 4 mm (*p* = 0.179). MHR values were significantly different (*p* < 0.001) at all depths except between 0 and 1 mm (*p* = 0.565).

### 3.3. Reduction in Microhardness Ratio

**Figure 4:**
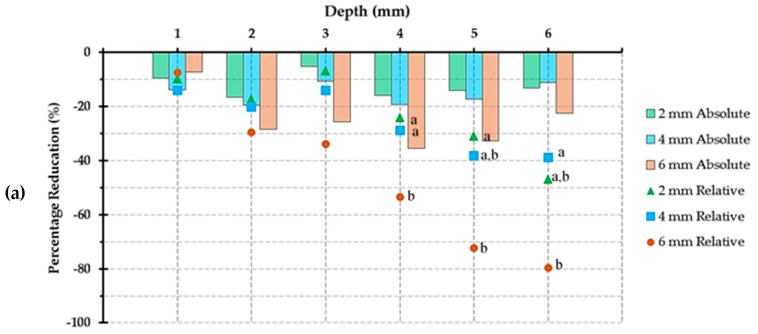

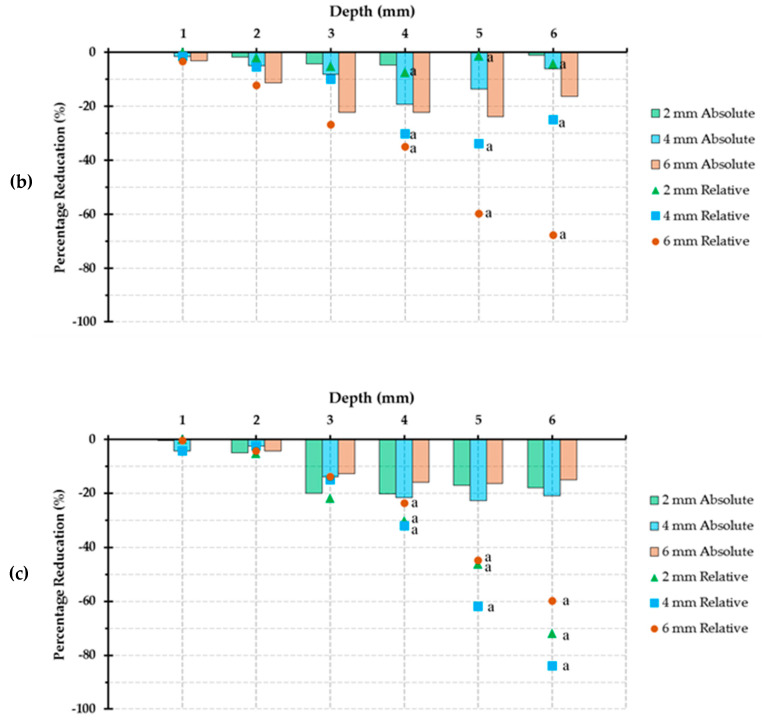
Bar graphs showing absolute reduction in microhardness ratio plus relative reduction in microhardness ratio (markers) compared to the same depth at 0 mm light cure distance for: (**a**) Tetric N-Ceram BF; (**b**) Filtek One BF, and; (**c**) Sonic Fill 2. The same letters indicate non-significant difference (*p* > 0.05) of relative reduction in microhardness ratio for a particular depth (showing values for 4, 5, and 6 mm depths).

Absolute and relative reductions in MHRs are included in (Figure 4). For TBF, the greatest values were 35.5% (LC_6_ at D_4_) and 79.7% (LC_6_ at D_6_) for absolute and relative reduction in MHR, respectively. For FBF, 23.8% (LC_6_ at D_5_) and 67.7% (LC_6_ at D_6_) were recorded for absolute and relative reduction in MHR, respectively. For SF2, the greatest values for absolute and relative reduction in MHR were 22.8% (LC_5_ at D_5_) and 84.0% (LC_4_ at D_6_), respectively.

### 3.4. Spectral Irradiance

**Figure 5:**
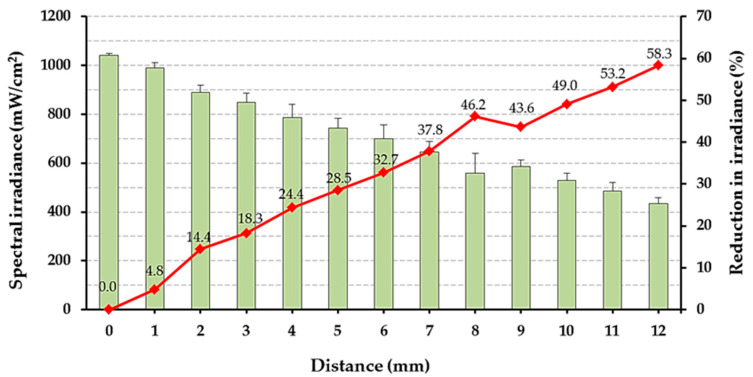
Spectral irradiance values (mW/cm^2^) for each distance (bars with error bars representing standard deviations) as well as percentage reduction in irradiance values (line with labels) compared to distance = 0 mm.

Figure 5 illustrates the spectral irradiance measured in mm increments. There is a marked decrease in the irradiance values from 0 to 12 mm.

## 4. Discussion

Bulk-fill dental resin composites manufacturers claim that these materials can be sufficiently cured up to 4–5 mm depth, when used with the same LC time with at least 1000 mW/cm^2^ irradiance [7,22]. Holding the LC tip away from the restoration surface is not uncommon in dental practice, as the restorative material placed at the floor of a deep cavity will be further from the LC tip than the remainder of the restoration. This can affect the restoration’s physical properties [12,13]. This study aimed to test the adequacy of cure of dental bulk-fill resin composites placed in a single increment of 4–5 mm at different depths and with varying distances from the LC tip. The null hypothesis was rejected since significant differences were found between groups of varying depth and LC distances.

The three BF resin composites that were chosen to be investigated in this project are commonly utilized in dental practices and from well-established manufacturers [23,24,25]. Vickers microhardness test is a well-established test for measuring the microhardness of composites [26,27,28]. MHR was chosen in order to provide a sense of the depth of cure (DoC) of the investigated materials at the tested conditions as well as the degree of conversion (DC). This is due to the fact that MHR was found to be strongly correlated to the DC [29], having a linear relationship [26].

The mold and spacers used to fabricate the specimens were made of stainless steel, which ensured that no scattered light would contribute to the polymerization of the specimen and only direct light would be allowed to pass through. In addition, the use of spacers allowed the standardization of LC distance among the tested groups. The area of interest for bulk-fill resin composite materials lay in their ability to be adequately cured at the claimed depth of 4–5 mm, thus the absolute and relative reduction in MHR for the tested materials was analyzed and compared at these depths and with LC_2_, LC_4,_ and LC_6_ relative to LC_0_. In a clinical setting, the actual distance may be much higher than 6 mm since some class II cavities are more than 6 mm in depth. In deep gingival seats of proximal cavities, the distance between LC tip and the floor of the cavity can reach 10 mm if the LC tip is not in direct contact with the tooth [30]. In addition, several LC distances were investigated, since not all practitioners ensure direct contact between the LC tip and the occlusal plane of the restored tooth. Specimens were stored for 1 week in order to ensure complete polymerization of the material used and provide maximum value for materials’ properties.

Significant reduction in curing efficiency started at 2 mm depth of the restoration with the tested materials in this project. The most probable reason for this is the decrease in irradiance at the 2 mm distance from the LC. As shown in Figure 5, the irradiance dropped to below 900 mW/cm^2^ at 2 mm. Most manufacturers recommend at least 1000 mW/cm^2^ for bulk-fill materials [7,22].

FBF and SF2 microhardness values were not affected significantly by the change in LC distance at 4-, 5- or 6-mm depths. For TBF, comparing 4-, 5- and 6-mm depths, microhardness values as well as relative reduction in MHR were drastically reduced with the increase in depth. This finding is in line with a study by Diab et al. wherein they found that the effect of LCD is material-dependent; they also reported lower MHR values for TBF when compared to FBF [18]. This could be due to inherent properties within TBF owing to its chemistry and the utilization of an additional photoinitiator (Ivocerin). However, further investigations must be conducted in order to verify this theory. Although the study by Diab et al. [18] reported similar findings to the current paper, the two reports were different in many regards: the depth of the tested specimens (6 mm vs. 4 mm depth), storage duration (one week vs. 24 h), the number of tested bulk-fill materials (SF2 was included in the present study), and the determination of the microhardness profile in the current report, which was not included in the 2021 paper.

The acceptable bottom-to-top MHR should be at least 0.8 for the material to be adequately cured [21]. TBF showed acceptable MHR values up to approximately 2.8, 2.0, 1.6 and 1.5 mm depths with LC_0_, LC_2_, LC_4_, and LC_6_, respectively (Figure 2). FBF showed acceptable MHR values up to approximately 3.3, 2.9, 2.6, and 2.1 mm depths with LC_0_, LC_2_, LC_4_, and LC_6_, respectively. SF2 showed acceptable MHR values up to approximately 3.5, 3.0, 2.6, and 2.5 depths with LC_0_, LC_2_, LC_4_, and LC_6_ respectively. None of the tested BF resin composite materials had sufficient MHR at the depths of 4,5, or 6 mm with any LC distance. It is interesting to report that at D_6_ with LC_6_, the MHRs for all the tested materials were 8, 10, and 4% for TBF, FBF, and SF2, respectively.

From the above findings, the best results were obtained with LC_0_ at D_0_. Increasing the LC distance and/or the depth of the restoration significantly affects the Vickers microhardness values and MHR. The effect of depth on the outcome is in line with many previous studies [2,16,18,20,31,32,33,34]. Restorative dentists must be aware of the significant effect of LC distance on the physical properties of the bulk-fill resin composite materials. None of the tested materials showed acceptable MHR beyond 3.5 mm depth. A recent systematic review presented the summary estimate from previous studies for the MHR of TBF, Sonic-Fill, SureFil, and Venus Bulk-fill and found that at 4 mm depth, most of the studies on the materials used in their study have acceptable 0.8 MHR, except for Sonic-Fill where most of the studies has non-acceptable MHR values [35]. Previous studies have also reported acceptable MHR for TBF up to 4 mm depth [16,22], while others have found the reported MHR to be not acceptable [20,36,37]. Some authors reported acceptable MHR for TBF and FBF at 4.88- and 5.63-mm depths, respectively, and all tested bulk-fill materials showed acceptable MHR at least at 4.5 mm depth [38]. This discrepancy may be due to differences in the used LC intensity level, duration, distance, and type (polywave vs. monowave).

There was no reduction in MHR for all three materials at D_0_ regardless of LC distances. Absolute and relative reduction in MHR for FBF and SF2 at D_1_ and LC_2_ is also zero. At D_4_, D_5_, and D_6_ with LC_6_, SF2 showed the least reduction in MHR among the tested materials, while TBF showed the highest values of reduction. This could be justified by the differences in composition of the tested materials and the utilization of different photoinitiator combination.

As with other in vitro investigations, this study has limitations. These include that the tested materials all used the same shades with the same LC unit to initiate polymerization. Using different materials shades and translucency levels [37], different LC type and duration, testing these materials at different time points after LC, with different LC tip angulation, light beam profile/distribution, photoinitiator, filler (type, size, volume), as well as storing them in an oral cavity simulating environment are all options for further testing to give a broader understanding of the different materials. More BF materials may also be tested using the same methodology for homogeneity and ability to compare the outcome. It was found that flowable BF materials perform superior to regular bulk-fill materials regarding the depth of cure [19,34,35,37,39], thus testing and comparing both types of BF materials is suggested. Additionally, including a control conventional resin composite material can give us a comparable outcome with BF resin materials, as in a study where they found that the Knoop hardness values for conventional Filtek Z350 is higher than the other tested two bulk-fill resin composites [40]. Investigating other outcomes indicating the efficiency of curing and thus performance of the bulk-fill resin materials such as the degree of conversion and remaining thickness would add valuable information. Investigating other contributing factors to these findings in future research is paramount. Additionally, further testing is required to ensure the chemistry-specific behavior of the BF materials.

From the above findings, it is clear that clinicians need to be aware of the thickness of the BF material increment and of the distance at which the LC depth is held from the surface and bottom of the cavity. Within the limitations of this study, it was found that the best outcome is obtained with the LC tip in direct contact with the restoration surface and with an increment of less than 3 mm thickness. Caution must be taken especially in deep proximal cavities in which depths of 5 mm, or more, are not uncommon. This brings the role of deep fillings on local inflammation into consideration. This is due to the fact that micro/nano-particles are released from deeply placed restorations such as implants and deep composite restorations [41]. A recent systematic review has found no conclusive evidence regarding the sealing capacity of bulk-fill materials in cavities with their margins in cementum [42]. Constant attention to oral health conditions, while using the appropriate materials, increases the longevity of the teeth in the oral cavity [43]. In addition, dental students and recently graduated practitioners must take into consideration the light cure parameters that can interfere with the efficient polyermization of BF materials.

## 5. Conclusions

Within the limitations of this in vitro study, it has been found that increasing the LC distance and/or the depth of the tested BF restorations significantly affects the Vickers microhardness values and MHR. Inadequate microhardness values of the BF materials can lead to clinical complications including accelerated wear, restoration fractures and secondary caries [5]. TBF has much lower microhardness values and MHR compared to FBF and SF2. None of the tested bulk-fill resin composite materials had sufficient MHR at the claimed depths of either 4, 5, or 6 mm. SF2 showed the least reduction in MHR.

The recommendation for the restorative dentist remains to place the LC tip as close to the restorative material as possible when during the polymerization process to optimize the material’s properties.

## Figures and Tables

**Table 1:** Composition of the materials used in the study.

Material	Main Monomer	Main Fillers	Photo-Initiator	Manufacturer
Filtek One Bulk-Fill (FBF)	Aromatic urethane dimethacrylate (AUDMA)	Silane-treated ceramics	Camphorquinone (CQ)	3M ESPE, Dental Products, Saint Paul, MN, USA
Tetric N-Ceram Bulk-Fill (TBF)	Bisphenolglycidyl methacrylate (Bis-GMA)	Barium aluminium silicate glass	Camphorquinone (CQ) and Ivocerin	Ivoclar Vivadent, Zurich, Switzerland
SonicFill 2 (SF2)	3-trimethoxysilylpropyl methacrylate	Barium glass	Camphorquinone (CQ)	Kerr Dental, Orange, CA, USA

**Table 2:** Means and standard deviations (SD) of Vickers microhardness values of the three bulk-fill materials investigated in the study.

		Ivoclar Tetric N-Ceram Bulk-Fill		3M Filtek One Bulk-Fill		Kerr Sonic-Fill 2	
LC Distance	Depth (mm)	Mean	SD	Mean	SD	Mean	SD
0 mm	0	17.33	1.36	23.17	1.04	23.09	0.42
	1	16.92	1.37	22.58	2.33	23.42	1.07
	2	16.59	0.79	21.42	2.53	22.67	1.09
	3	13.17	1.75	19.17	4.13	21.25	1.66
	4	11.50	0.79	14.67	2.55	15.42	3.40
	5	7.92	1.26	9.17	3.10	8.42	5.32
	6	4.83	1.14	5.50	2.86	5.75	6.70
2 mm	0	18.17	0.43	22.67	0.72	23.58	1.20
	1	16.00	0.27	22.08	0.74	24.09	1.07
	2	14.50	1.55	20.50	2.40	22.00	0.98
	3	12.92	1.29	17.75	2.54	17.00	1.79
	4	9.25	1.26	13.42	3.35	11.00	2.76
	5	5.67	1.19	9.00	3.67	4.58	1.73
	6	2.75	0.96	5.25	2.32	1.67	0.47
4 mm	0	17.50	0.84	23.17	0.88	24.92	1.90
	1	14.75	2.13	22.17	0.69	24.00	1.41
	2	13.50	2.69	20.33	0.72	23.75	0.69
	3	11.50	1.00	17.25	1.93	19.42	0.96
	4	8.25	1.14	10.42	3.94	11.17	3.19
	5	4.92	1.53	6.17	2.78	3.42	1.97
	6	3.00	0.82	4.17	1.50	1.00	0.00
6 mm	0	17.92	0.57	22.75	0.57	23.00	1.22
	1	16.25	1.07	21.42	1.71	23.83	2.70
	2	12.17	1.04	18.50	1.99	20.42	2.17
	3	9.08	1.10	13.75	0.42	17.08	1.52
	4	5.50	0.43	9.33	4.22	10.42	1.37
	5	2.25	0.78	3.59	2.20	2.50	1.04
	6	1.00	0.00	1.75	1.50	1.00	0.00

Tetric N-Ceram BF was significantly different from Filtek One BF and Sonic-Fill 2 (*p* ≤ 0.05). Microhardness values were significantly different (*p* ≤ 0.05) at all light cure distances except between 2 and 4 mm. Microhardness values were significantly different (*p* ≤ 0.05) at all depths except between 0 and 1 mm.

## Data Availability

Not applicable.
